# Carnitine Deficiency in an Adult With Short Bowel Syndrome After Surgical Resection

**DOI:** 10.14309/crj.0000000000000799

**Published:** 2022-06-20

**Authors:** Amir Y. Kamel, Nicole C. Ruiz, Melissa R. Murray, Ansley M. Gayle, Angela Pham, Thiago Beduschi, Martin D. Rosenthal

**Affiliations:** 1 Department of Pharmacy, University of Florida Health Hospital, Gainesville, FL; 2 Department of Internal Medicine, University of Florida Health Hospital, Gainesville, FL; 3 Division of Gastroenterology, Hepatology & Nutrition, Department of Medicine, University of Florida Health Hospital, Gainesville, FL; 4 Division of Transplantation & Hepatobiliary Surgery, Abdominal Transplant Program, Department of Surgery, University of Florida Health Hospital, Gainesville, FL; 5 Department of Surgery, Abdominal Wall Reconstruction and Intestinal Rehab Service (UFAIR), University of Florida Health Hospital, Gainesville, FL

## Abstract

Carnitine is an essential cofactor for fatty acid metabolism. Deficiencies can be associated with muscle weakness, fatigue, weight loss, and cardiomyopathies. A 27-year-old woman with short bowel syndrome (SBS) presented with significant weight loss, fatigue, and muscle wasting despite adequate parenteral nutrition. Her laboratory test results revealed carnitine deficiency secondary to malnutrition. Levocarnitine supplementation was initiated with normalization of her carnitine levels. Her fatigue improved, and her weight returned to baseline. Carnitine deficiencies are seldomly reported in adults, particularly those with SBS. Carnitine deficiency should be suspected and corrected in patients with SBS and malabsorptive capacity due to surgical resection.

## INTRODUCTION

Carnitine is essential for fatty acid-mediated energy metabolism and branch chain amino acid catabolism.^1^ It functions in the transport of long-chain fatty acids from cytosol to the mitochondrial matrix and facilitates the incorporation of fatty acids into phospholipids during oxidative repair.^2^ Carnitine also maintains mitochondrial integrity by blocking the formation of reactive oxygen species.^3^ This amino acid derivative is obtained from diet and endogenous biosynthesis.^1^ Carnitine deficiencies can interfere with the metabolism of macronutrients and manifest as muscle weakness, fatigue, vomiting, abdominal pain, weight loss, hypoglycemia, metabolic acidosis, neurological deficits, or cardiomyopathies. Carnitine deficiencies are not widely reported in adults, particularly those with short bowel syndrome (SBS). Many cases are asymptomatic, remain undiagnosed, and are untreated.^4^

## CASE REPORT

A 27-year-old White woman with a history of fatty liver, severe dysmotility, and intestinal malrotation with recurrent complete small bowel obstructions complicated by extensive peritoneal adhesions had multiple hospital admissions secondary to malnutrition and small bowel obstruction leading to intestinal ischemia.

She underwent multiple bowel resections, was left with 150 cm of tapered jejunum anastomosed to the rectum, and met diagnostic criteria for SBS.^5^ Six months after surgery, she continued to have significant fatigue, moderate bilateral muscle wasting, myopathy, loss of subcutaneous fat stores, and a 3 kg weight loss (54–51 kg) despite intake of 1,640 calories per day through exclusive parenteral nutrition (PN). Index micronutrient levels were drawn (Table 1). Her carnitine ester/free ratio was normal ruling out excess renal loss of carnitine.^2^ Both free and total carnitine levels were found to be markedly low (Table 1). Liver function tests, thyroid function tests, triglycerides, C-reactive protein (CRP), erythrocyte sedimentation rate (ESR), creatinine kinase, cortisol, beta-hydroxybutyrate, and glucose levels were within a normal range. Levocarnitine (2,000 mg), pyridoxine (50 mg), additional protein, and carbohydrates were supplemented in her PN for 6 months with improvement in energy levels and weight gain (49.9–58.1 kg).

**Table 1.** Serum micronutrient concentrations in a 27-year-old woman and corresponding reference ranges

Nutrient	Index serum micronutrient concentration	Normal range	Interpretation	Repeat concentration at 8 mo	Repeat concentration at 20 mo
Carnitine free, µmol/L	9	25–60	Low	8	69
Carnitine total, µmol/L	12	34–86	Low	10	87
Carnitine ester/Free ratio	0.3	0.1–1.0	Normal	0.2	0.7
Folate, ng/mL	18	4.4–19.9	Normal	17	N/A
Thiamine (vitamin B1), nmol/L	199	70–180	High	N/A	N/A
Pyridoxine (vitamin B6), nmol/L	21.5	20–125	Normal	24.4	42.5
Cyanocobalamin (vitamin B12), pg/mL	416	243–894	Normal	>2,000	861
Zinc, µg/dL	34	60–120	Low	84	132
Selenium, mcg/L	135	63–160	Normal	N/A	N/A
Copper, µg/dL	78	80–155	Low	N/A	174

She attempted exclusive enteral nutrition for 8 months but continued to experience chronic fatigue and weight loss (63.8–48.8 kg) and was restarted on exclusive PN with levocarnitine supplementation. Repeat serum micronutrient concentrations were subtherapeutic with a free carnitine level of 8 μmol/L and a total level of 10 μmol/L (Table 1). A citrulline level was ordered to determine the functional status of her remaining bowel. Her citrulline level was 15 μmol/L (normal range 20–50 μmol/L), indicating chronic intestinal failure with a maladaptive capacity.^6^ One year after reinitiating PN with levocarnitine supplementation, the patient's carnitine and micronutrient levels increased, she gained approximately 10 kg (48.8–58.4 kg), and her energy levels improved (Table 1).

She underwent evaluation for a primary carnitine deficiency, which was ruled out by the acylcarnitine panel. In the setting of normal renal function, normal urinalysis with no ketones and only trace protein, and no pharmacological agents that cause carnitine deficiency, her deficiency was attributed to malnutrition from SBS.

## DISCUSSION

Carnitine and acetylcarnitine are essential metabolites for oxidative phosphorylation and energy production. Physiologic homeostasis of carnitine relies on absorption from diet, endogenous biosynthesis, and efficient renal reabsorption.^7^ Deficiencies vary in clinical presentation, ranging from fatigue or weight loss to hepatic dysfunction, renal failure, or death. The carnitine status is determined by intestinal carnitine absorption, tissue uptake, endogenous synthesis, and renal reabsorption.^8^ Although cardiac and skeletal muscles have high concentrations of carnitine, they lack the ability to synthetize carnitine.^9,10^ Primary carnitine deficiencies occur because of genetic abnormalities leading to a defective carnitine transporter or impaired endogenous biosynthesis.^11^ Secondary deficiencies occur from increased renal tubular loss of free carnitine, increased excretion from pharmacologic therapy, depleted stores, inherited metabolic disorders, or malnutrition.^1^ Although 75% of carnitine is derived from diet, endogenous production of carnitine is usually sufficient to meet physiologic needs.^2^ Strict vegetarians obtain more than 90% of carnitine through biosynthesis, yet deficiencies have not been reported.^11^

In this patient, investigation for carnitine deficiency was triggered by signs and symptoms including a history of fatty liver, chronic fatigue, bilateral muscle wasting, rapid weight loss, myopathy, and loss of subcutaneous fat stores despite adequate nutritional support. Evaluation for a primary carnitine disorder was negative, and she was diagnosed with a secondary deficiency caused by inadequate exogenous carnitine in the setting of SBS.

Citrulline functions as a surrogate marker of intestinal failure because it is almost exclusively dependent on intestinal synthesis. Levels below 20 μmol/L in SBS are associated with chronic intestinal failure, impaired macronutrient and micronutrient absorption, and requirement of long-term PN.^6^ Endogenous production of carnitine requires lysine and methionine precursors for biosynthesis.^12^ In the index case, an amino acid assay was not initially ordered. After PN, subsequent panels showed normal lysine and methionine levels with low citrulline levels as expected in SBS. The patient showed weight gain and increased energy levels after receiving levocarnitine-supplemented PN.

Carnitine deficiencies in SBS are rare. Reports by Miyajima et al and Hirose et al regarding patients with SBS found to have carnitine deficiencies secondary to inadequate dietary intake that were supplemented with levocarnitine. The 64-year-old patient in the study of Miyajima et al experienced carnitine deficiency 10 months after bowel resection.^13,14^ A similar timeline has been seen in patients undergoing jejuoileostomy.^15^ However, studies have shown that PN-dependent SBS children with no enteral feeding can develop carnitine deficiency within weeks despite having the ability to endogenously synthesize carnitine.^16–18^ It has been suggested that monitoring of plasma carnitine should be considered for children on PN greater than 4 weeks.^19^

Other micronutrient deficiencies commonly seen in PN-dependent patients include zinc, copper, selenium, and pyridoxine. Zinc and pyridoxine deficiencies are seen in high gastrointestinal loss states; zinc deficiency manifests as skin and eye lesions or alopecia while pyridoxine deficiency causes peripheral neuropathy, dermatitis, and glossitis. Copper is often removed from PN in cholestasis; signs and symptoms of the deficiency include myelopathy, ataxia, and paresthesia. Selenium deficiency has been reported in home PN patients without supplementation and presents as infertility and myopathy.^18^

In conclusion, levocarnitine PN supplementation in an adult SBS female patient after bowel resection resulted in improved nutritional status, weight loss, and fatigue. Malnutrition as a consequence of SBS was likely the cause of deficiency. It is important to consider trace mineral or vitamin deficiencies in patients with SBS.

## DISCLOSURES

Author contributions: AY Kamel wrote the article and is the article guarantor. NC Ruiz edited the article, reviewed the literature, and provided the images. MR John and AM Gayle revised the article for intellectual content. All authors contributed to the approval of the final article.

Financial disclosures: None to report.

Informed consent was obtained for this case report.
